# Partially defatted rather than native poppy seeds beneficially alter lipid metabolism in rats fed a high-fat diet

**DOI:** 10.1038/s41598-023-40888-x

**Published:** 2023-08-29

**Authors:** Jarosław Koza, Adam Jurgoński

**Affiliations:** 1 Department of Gastroenterology and Nutrition Disorders, Faculty of Health Sciences, Collegium Medicum in Bydgoszcz, Nicolaus Copernicus University in Toruń, Ujejskiego 75 Str., 85-168 Bydgoszcz, Poland; 2 Department of Biological Function of Food, Institute of Animal Reproduction and Food Research, Polish Academy of Sciences, Tuwima 10 Str., 10-748 Olsztyn, Poland

**Keywords:** Nutrition disorders, Obesity, Fatty acids, Oils, Metabolic syndrome, Obesity, Metabolism, Dyslipidaemias, Non-alcoholic steatohepatitis

## Abstract

Partially defatted poppy seeds, a by-product of poppy oil cold pressing, could be an interesting dietary supplement for obesity management. The aim of this study was to compare the effects of dietary supplementation with a small amount of native or partially defatted poppy seeds on gastrointestinal function and lipid metabolism in rats fed a high-fat diet. The defatted poppy seeds had, among others, lower fat content and higher fibre and protein content than native poppy seeds. The rats fed with a high-fat diet were characterised by severe metabolic disorders, especially in the liver, and poppy seeds were unable to prevent them. However, depending on the seed form, dietary supplementation with poppy seeds differentially affected the microbial and endogenous lipid metabolism in rats. In the distal intestine, both dietary seed forms stimulated microbial acetate production, and the supplementation with partially defatted poppy seeds additionally inhibited isobutyrate and isovalerate formation, which indicates a reduction in putrefaction. Both dietary seed forms increased cholesterol accumulation in the liver. Only dietary supplementation with partially defatted poppy seeds attenuated visceral fat and hepatic triglyceride accumulations and lowered blood triglyceride concentrations, and at the transcriptional level, the inhibition of *SREBP-1c*, which upregulates genes responsible for de novo lipogenesis, was additionally observed in this organ. In conclusion, a low and regular consumption of partially defatted poppy seeds may be beneficial in managing obesity-related disorders.

## Introduction

Opium poppy (*Papaver somniferum* L.) is a flowering plant widely known for its culinary and pharmacological uses. Opium poppy contains specific alkaloids, including the most known morphine, obtained from the seed capsules of this plant^1,2^. Poppy seeds, in turn, are the source of nutrients and other bioactive compounds^3^ and usually do not contain alkaloids, unless contaminated with them^4,5^. Poppy seeds meet consumers’ preferences and are eaten in various forms, either whole or ground, and added to retailed food products or regional dishes, such as poppy seed noodles or pastes. Bread, sweets and confectionery products, such as cakes, pancakes, muffins or cookies, may contain poppy seeds in different quantities, from a small decorative addition to bread to the main ingredient of poppy seed cake (makowiec in Polish). The popularity and use of poppy seeds varies depending on the part of the world, for example poppy seed noodles are a traditional dish in Poland and other Central European counties, and poppy seed pastes are made in Turkey and other Western Asian countries^6^.

The main components of poppy seeds are fat (40–50%), dietary fibre (20–30%), mainly its insoluble fraction, and protein (10–20%)^7,8^. The oil extracted from the seeds predominantly contains polyunsaturated fatty acids (PUFAs) with linoleic acid (18:2 *n−*6) as the main fatty acid (60–70%)^7, 8^. The other fatty acids present in the oil in larger quantities are palmitic acid (14%) and oleic acid (11%) that represent saturated and monounsaturated fatty acids (SFAs and MUFAs), respectively. The protein quality of the seeds is relatively high containing all essential amino acids with tryptophan as the limiting amino acid^8^. Poppy seed oil also contains a significant amount of tocopherols (up to 340 mg/kg), including mainly *γ*-tocopherol, some amount of phytosterols (approx. 2 mg/kg), and volatile compounds^7, 9^. Moreover, phenolic compounds, including flavonoids, were also detected in poppy seeds^8,9^.

Cold-pressed vegetable oils are becoming more and more popular due to their minimal processing, which is considered the basis of a healthy diet, and poppy seed oil also fits into this trend^10^. During cold pressing, a by-product in the form of defatted poppy seeds is obtained and suggested as a valuable and safe source of nutrients and biologically active compounds for further potential use in the food and pharmaceutical industries^8,11^. However, despite interesting composition of poppy seeds, there is a lack of knowledge about health consequences of their consumption in both native and defatted form. Moreover, previous studies indicate that the non-lipid fraction of certain seeds is largely responsible for attenuating obesity-related metabolic disorders, including those of the distal intestine and lipid metabolism, which is due to dietary fibre and/or other seed-specific bioactive compounds^12, 13^. Thus, the aim of the present study was to compare the effects of dietary supplementation with a small amount of native or defatted poppy seeds on gastrointestinal function and lipid metabolism in rats fed with a high-fat diet. We hypothesised that consuming defatted poppy seeds could be beneficial to rats due to a wide variety of components other than those pressed with the oil.

## Results

### Chemical composition of poppy seeds

The composition of native poppy seeds and defatted poppy seeds, which was a by-product in the production of cold-pressed oil, is shown in Table 1. The dry matter content was higher in native poppy seeds than in defatted poppy seeds (94% vs. 91%), whereas the ash content was lower in native poppy seeds than in their defatted form (7% vs. 10%). The main components of both seed forms were fat, protein and fibre. Native poppy seeds were especially rich in fat (44%), and the protein and fibre content in these seeds was 21% and 20%, respectively. The defatted form of poppy seeds had a higher fibre and protein content (33% and 27%, respectively), but still contained a significant amount of fat (21%) due to cold pressing, which is not as efficient as other methods, such as hot pressing or chemical extraction^14^. The fatty acid profile calculated as percentage of the fat fraction also differed significantly between the two seed forms (Table 1). Native poppy seeds had a higher percentage of PUFAs than their partially defatted form (66% vs. 41%) with linoleic acid being the main fatty acid in both seed forms (65% and 41%, respectively). As a consequence, the defatted poppy seeds had a higher percentage of SFAs and MUFAs. The SFA percentage was 16% in partially defatted poppy seeds and 12% in native poppy seeds with palmitic acid as the main fatty acid in both seed forms (16% and 10%, respectively). The MUFA content was 38% in partially defatted poppy seeds and 17% in native poppy seeds, including oleic acid as the main fatty acid in both seed forms (38% and 16%, respectively). Native poppy seeds also contained a small percentage of other fatty acids, which were absent in their partially defatted form (Table 1).

**Table 1:** Chemical composition of native and partially defatted poppy seeds.

	Native poppy seeds	Partially defatted poppy seeds
Dry matter, %	94.0 ± 0.09	90.7 ± 0.10
Ash, %	7.21 ± 0.03	9.85 ± 0.04
Dietary fibre, %	20.3 ± 0.25	32.6 ± 1.45
Nitrogen-free extract, %	1.32 ± 0.01	0.08 ± 0.01
Crude protein, %	21.1 ± 0.06	27.1 ± 0.41
Crude fat, %	43.8 ± 0.17	20.9 ± 0.27
Fatty acid profile (%)		
Palmitic acid (16:0)	9.88 ± 0.03	16.3 ± 4.05
Palmitoleic acid (16:1 *n−*7)	0.11 ± 0.00	–
Stearic acid (18:0)	2.27 ± 0.00	–
Oleic acid (18:1 *n−*9)	15.5 ± 0.03	38.2 ± 1.90
Vaccenic acid (18:1 *n−*7)	1.38 ± 0.01	–
Linoleic acid (18:2 *n−*6)	64.9 ± 0.06	40.7 ± 5.92
Arachidic acid (20:0)	0.85 ± 0.00	–
Gondoic acid (20:1 *n−*9)	0.10 ± 0.00	–
α-Linolenic acid (18:3 *n−*3)	0.65 ± 0.00	–
Docosahexaenoic acid (22:6 *n−*3)	0.17 ± 0.00	–
Others	0.20 ± 0.00	–
Calculated fatty acid content (%)		
SFAs	12.3 ± 0.03	16.3 ± 4.05
MUFAs	17.1 ± 0.04	38.2 ± 1.90
PUFAs	65.7 ± 0.06	40.7 ± 5.92
*n*-3	0.82 ± 0.00	–
*n*-6	64.9 ± 0.09	40.7 ± 5.92

All values are expressed as the mean ± SD (*n* = 3). SFAs, saturated fatty acids; MUFAs, monounsaturated fatty acids; PUFAs, polyunsaturated fatty acids.

### Dietary intake, body weight and body composition of rats

To create an animal model of obesity-related disorders, a high-fat diet was used for 8 weeks in rats (group HF, positive control), which was additionally supplemented with 1% of native poppy seeds or partially defatted poppy seeds (HF + PS or HF + DPS group, respectively, see Table 2 and the “Methods” section for details). The initial body weight of rats was comparable among the control (regular fat content in the diet), HF, HF + PS and HF+DPS groups, as was the initial body fat and lean percentage (Table 3). During 8 weeks of experimental feeding, the dietary intake was 12% lower in the HF groups compared to the C group, and the dietary supplementation with poppy seeds, regardless of their form, had no effect on it. Despite the lower dietary intake, rats from each HF group gained more body weight, including body fat mass, compared to the C group. The final body weight was 10% higher in the HF group than in the C group. After 8 weeks of feeding, the final body fat percentage was also higher in all HF groups, whereas the final body lean percentage was lower, compared to the C group. However, the epididymal fat percentage, which is an important part of visceral fat in rodents^15^, was significantly higher only in the HF and HF + PS group compared to the C group (approx. by 20%). In the HF + DPS group, the epididymal fat percentage was comparable to that of the C and HF group (Table 3).

**Table 2:** Composition of the diets (g/100 g).

	Group			
	C	HF	HF + PS	HF + DPS
Ingredients				
Casein	20	20	20	20
DL-methionine	0.3	0.3	0.3	0.3
Rapeseed oil (canola type)	7	7	7	7
Palm oil	–	18	18	18
Cholesterol	–	0.5	0.5	0.5
Native poppy seeds	–	–	1	–
Partially defatted poppy seeds	–	–	–	1
Corn starch	53.0	34.3	33.5	33.5
Sucrose	10	10	10	10
Cellulose	5.0	5.0	5.0	5.0
Mineral mix	3.5	3.5	3.5	3.5
Vitamin mix	1	1	1	1
Choline chloride	0.2	0.2	0.2	0.2
Components				
Protein	18	18	18	18
Fat	7	25	25	25
Fibre	5.5	5.5	5.5	5.6

C, control group; HF, high-fat group; HF + PS, high-fat group supplemented with native poppy seeds; HF + DPS, high-fat group supplemented with partially defatted poppy seeds. Chemical composition of casein (g/100 g): crude protein, 87.0; crude fat, 0.30; dietary fibre not detected; ash, 2.0. Chemical composition of poppy seeds is shown in Table 1. The mineral and vitamin mixtures were in accordance with the recommendations of the AIN-93 diet^33^.

**Table 3:** Effects of dietary supplementation with native or partially defatted poppy seeds on body weight, diet intake and body composition of rats.

	Group				ANOVA *P* value
	C	HF	HF + PS	HF + DPS	
Initial body weight (g)	151 ± 2.48	155 ± 1.74	156 ± 3.01	153 ± 4.07	NS
Initial fat (%)	16.2 ± 0.259	17.8 ± 0.559	17.9 ± 0.696	17.7 ± 1.27	NS
Initial lean (%)	71.3 ± 0.670	69.8 ± 0.461	70.1 ± 1.25	69.6 ± 1.48	NS
Dietary intake (g/day)	19.4 ± 0.220^a^	17.1 ± 0.286^b^	17.1 ± 0.442^b^	16.8 ± 0.306^b^	< 0.001
Final body weight (g)	410 ± 6.66^b^	450 ± 9.38^a^	444 ± 9.61^a^	444 ± 11.0^a^	< 0.05
Final fat (%)	28.6 ± 1.23^b^	34.9 ± 1.21^a^	34.5 ± 0.89^a^	34.8 ± 1.32^a^	< 0.005
Epididymal fat (%)	3.64 ± 0.181^b^	4.46 ± 0.192^a^	4.24 ± 0.221^a^	4.20 ± 0.202^ab^	< 0.05
Final lean (%)	59.8 ± 1.15^a^	53.5 ± 0.91^b^	54.9 ± 0.99^b^	53.6 ± 0.98^b^	0.001
Body weight gain (g)	260 ± 7.50^b^	295 ± 8.97^a^	287 ± 9.37^a^	292 ± 9.77^a^	< 0.05
Fat gain (g)	92.9 ± 5.56^b^	130 ± 6.58^a^	125 ± 6.87^a^	128 ± 5.97^a^	0.001
Lean gain (g)	138 ± 6.09	132 ± 4.44	134 ± 4.62	132 ± 5.73	NS

Values are means ± SEMs (*n* = 7). Labelled means in a row without a common letter (a, b) differ at *P* ≤ 0.05 (Duncan’s or Dunn’s post hoc test). C, control group; HF, high-fat group; HF + PS, high-fat group supplemented with native poppy seeds; HF + DPS, high-fat group supplemented with partially defatted poppy seeds; NS, non-significant.

### Markers of gastrointestinal function in rats

Effects of dietary supplementation with native or partially defatted poppy seeds on gastrointestinal function is shown in Table 4. In the small intestine of all HF groups, the activity of mucosal disaccharidases (maltase and sucrase) was approximately two times lower than in the C group, apparently due to the lower content of carbohydrates in the diet of HF groups (details in Table 2). However, poppy seeds did not affect the mucosal disaccharidase activity. The main site for microbial fermentation of indigestible dietary components and thus for production of short-chain fatty acids (SCFAs) in rodents is the caecum^16^. Because the high-fat diet in the present study contained significantly less carbohydrates for microbial fermentation, the SCFA concentrations in the caecal digesta (each individual and total) were lower in the HF group compared to the C group (Table 4). Dietary supplementation with 1% poppy seeds affected the individual concentration of SCFAs to some extent, but did not change their total concentration. The acetate concentration, which is the main SCFA formed in the distal intestine^17^, was higher in the HF + PS and HF + DPS groups compared to the HF group, and at the same time it was comparable with that of the C group. The concentrations of branched SCFAs (isobutyrate and isovalerate) were lower in the HF and HF + PS group than in the C group, but the lowest concentrations of these fatty acids were in the HF + DPS group. The SCFA percentages in the caecal digesta also differed among groups (Table 4). The acetate percentage was higher, and the butyrate percentage was lower in all HF groups compared to the C group. The lowest total percentage of branched SCFAs (isobutyrate and isovalerate) was in the HF + DPS group, then it was higher in the HF and HF + PS group, and the highest—in the C group. All the aforementioned differences in the caecal formation of SCFAs were accompanied by a slightly and significantly lower pH value of the digesta in the HF + PS and HF + DPS groups, respectively, compared to the HF group (Table 4).

**Table 4:** Effects of dietary supplementation with native or partially defatted poppy seeds on gastrointestinal function in rats.

	Group				ANOVA *P* value
	C	HF	HF + PS	HF + DPS	
Small intestine					
Mass (g/100 g b.wt)	2.81 ± 0.108	3.08 ± 0.120	3.05 ± 0.069	2.96 ± 0.080	NS
pH of digesta	7.55 ± 0.088	7.50 ± 0.166	7.19 ± 0.141	7.50 ± 0.055	NS
Sucrase (µmol/min/g)	6.08 ± 1.17^a^	3.04 ± 0.42^b^	2.77 ± 0.65^b^	2.82 ± 0.40^b^	< 0.01
Maltase (µmol/min/g)	49.6 ± 6.10^a^	27.4 ± 4.21^b^	19.7 ± 4.02^b^	24.1 ± 2.72^b^	< 0.001
Caecum					
Mass of empty segment (g/100 b.wt)	0.159 ± 0.006	0.149 ± 0.006	0.152 ± 0.007	0.143 ± 0.004	NS
Digesta mass (g/g tissue)	3.85 ± 0.305	3.23 ± 0.263	3.25 ± 0.335	3.37 ± 0.275	NS
pH of digesta	7.70 ± 0.046^ab^	7.78 ± 0.030^a^	7.70 ± 0.095^ab^	7.57 ± 0.031^b^	< 0.05
Ammonia (mg/g digesta)	0.249 ± 0.015	0.252 ± 0.018	0.224 ± 0.018	0.235 ± 0.010	NS
SCFAs (µmol/g digesta)					
Acetate	40.6 ± 2.27^a^	31.5 ± 2.16^b^	35.7 ± 2.27^a^	34.9 ± 1.15^a^	< 0.05
Propionate	11.8 ± 0.647^a^	7.85 ± 0.678^b^	7.65 ± 0.637^b^	7.73 ± 0.686^b^	< 0.001
Isobutyrate	1.51 ± 0.063^a^	0.921 ± 0.091^b^	0.880 ± 0.059^b^	0.635 ± 0.066^c^	< 0.001
Butyrate	7.43 ± 0.521^a^	3.75 ± 0.617^b^	3.54 ± 0.364^b^	4.09 ± 0.576^b^	< 0.001
Isovalerate	1.53 ± 0.078^a^	0.957 ± 0.071^b^	0.905 ± 0.037^b^	0.631 ± 0.044^c^	< 0.001
Valerate	1.28 ± 0.184^a^	0.859 ± 0.089^b^	0.777 ± 0.112^b^	0.671 ± 0.082^b^	0.01
Total SCFAs	64.1 ± 3.28^a^	45.8 ± 3.52^b^	49.4 ± 2.63^b^	48.7 ± 1.71^b^	0.001
SCFAs (% total)					
Acetate	63.2 ± 0.891^b^	69.1 ± 1.27^a^	72.0 ± 1.86^a^	71.8 ± 1.32^a^	< 0.001
Propionate	18.4 ± 0.599	17.1 ± 0.479	15.5 ± 1.07	15.8 ± 1.05	NS
Butyrate	11.6 ± 0.677^a^	7.93 ± 0.763^b^	7.24 ± 0.703^b^	8.40 ± 1.10^b^	0.005
Branched SCFAs	4.80 ± 0.301^a^	4.10 ± 0.143^b^	3.65 ± 0.221^b^	2.59 ± 183^c^	< 0.001

Values are means ± SEMs (*n* = 7). Labelled means in a row without a common letter (a, b, c) differ at *P* ≤ 0.05 (Duncan’s or Dunn’s post hoc test). C, control group; HF, high-fat group; HF + PS, high-fat group supplemented with native poppy seeds; HF + DPS, high-fat group supplemented with partially defatted poppy seeds, NS, non-significant, SCFAs, short-chain fatty acids. Branched SCFAs are the sum of isobutyrate and isovalerate.

### Lipid metabolism and liver function in rats

**Figure 1:**
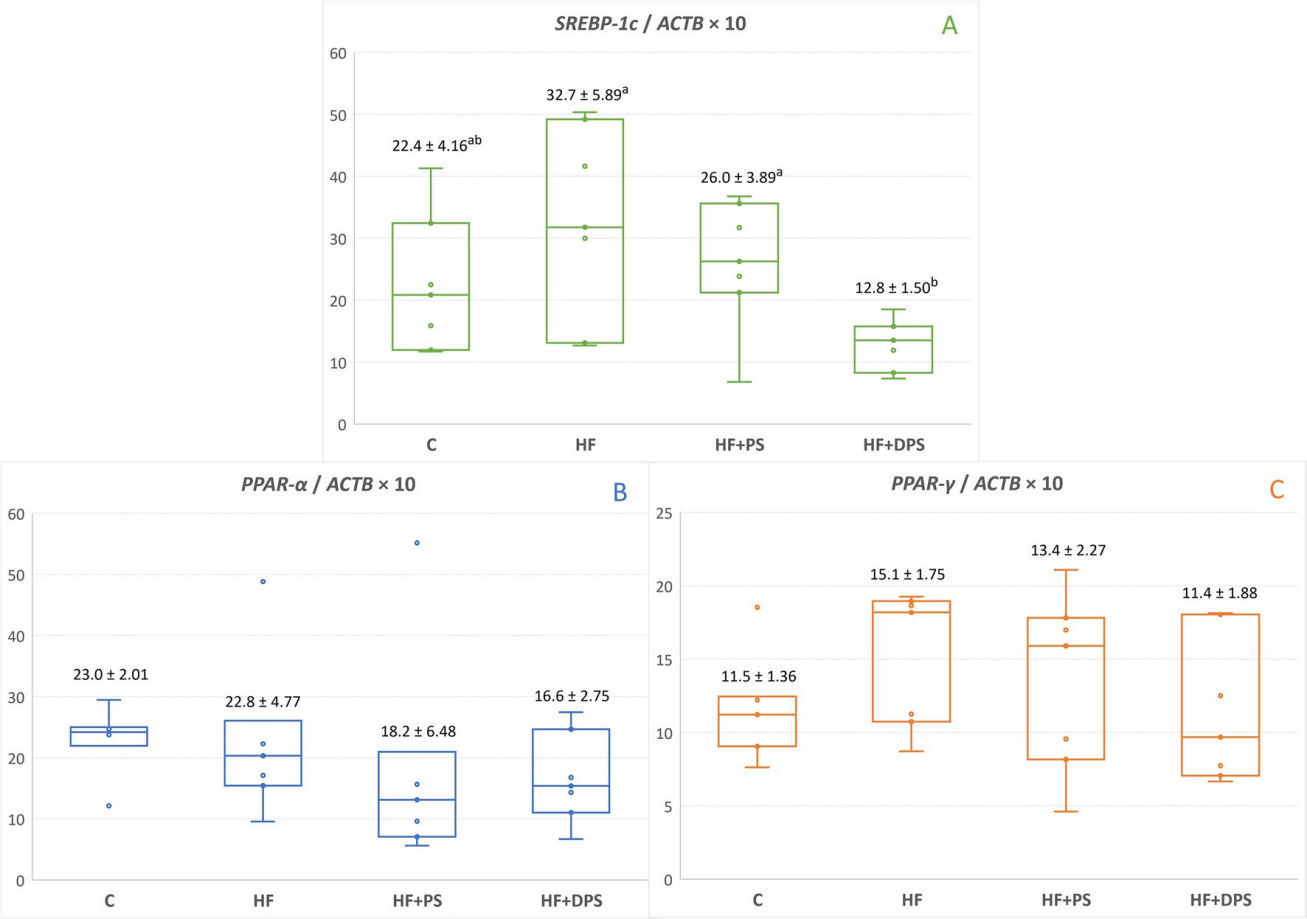
Effects of dietary supplementation with native or partially defatted poppy seeds on hepatic mRNA levels of transcription factors involved in lipid metabolism in rats. *PPAR-α*, peroxisome proliferator-activated receptor alpha gene (**A**); *PPAR-γ*, peroxisome proliferator-activated receptor gamma gene (**B**); *SREBP-1c*, sterol regulatory element-binding protein 1c gene (**C**); *ACTB*, beta-actin gene. The graph shows a box-and-whisker plot for the measurements in each group. The values above the bars are the means ± SEMs (*n* = 7). Labelled means without a common letter (a, b) differ at *P* ≤ 0.05 (Duncan’s or Dunn’s post hoc test). C, control group; HF, high-fat group; HF + PS, high-fat group supplemented with native poppy seeds; HF + DPS, high-fat group supplemented with partially defatted poppy seeds.

A high-fat diet used in the present study significantly affected endogenous lipid metabolism in rats. The liver relative mass, fat percentage, cholesterol and triglyceride concentration were higher in the HF group than in the C group (Table 5). These differences concerned especially liver lipids (total fat, cholesterol and triglycerides), the concentrations of which were several times higher in the HF group than in the C group. In addition, the mRNA level of sterol regulatory element-binding protein 1c gene (*SREBP-1c*) in the liver, which is a transcription factor responsible for stimulating hepatic *de novo* lipogenesis^18^, was also higher in the HF group than in the C group (Fig. 1A). This was not the case for hepatic mRNA levels of other genes encoding transcription factors involved in lipid metabolism (*PPAR-α* and *PPAR-γ*; Fig. 1B,C). A high-fat diet had relatively less effect on the blood lipid profile and only the non-HDL cholesterol concentration was higher in the HF group than in the C group (Table 5). Dietary supplementation with partially defatted poppy seeds rather than with their native form attenuated some of the lipid disorders induced by a high-fat diet, but due to their severity, they were still present in rats. The plasma triglyceride concentration was lower in the HF+DPS group than in the other groups (by 30% than in the HF+DPS group). The hepatic triglyceride concentration and *SREBP-1c* mRNA level were 14% and 61% lower, respectively, in the HF + DPS group than in the HF group. However, the liver cholesterol concentration was approx. 48% higher both in the HF + PS and HF + DPS group compared to the HF group. The liver malondialdehyde (MDA) concentration, which is a product of PUFA peroxidation^19^, did not differ among research groups. Moreover, the activity of certain liver enzymes in the blood plasma was higher in the HF group than in the C group (ALT and ALP, Table 5), and in the HF+DPS group, the alanine transaminase (ALT) activity was comparable to that of the C and HF group. The plasma total bile acid and glucose concentrations did not differ among research groups (Table 5).

**Table 5:** Effects of dietary supplementation with native or partially defatted poppy seeds on lipid metabolism and liver function in rats.

	Group				ANOVA *P* value
	C	HF	HF + PS	HF + DPS	
Liver					
Mass (g/100 b.wt)	3.49 ± 0.117^b^	4.86 ± 0.096^a^	4.88 ± 0.180^a^	4.91 ± 0.159^a^	< 0.001
Fat (%)	8.63 ± 0.728^b^	36.3 ± 2.902^a^	30.9 ± 1.62^a^	34.7 ± 1.59^a^	< 0.001
Cholesterol (mg/g)	1.21 ± 0.114^c^	7.21 ± 0.264^b^	10.7 ± 0.595^a^	10.8 ± 0.716^a^	< 0.001
Triglycerides (mg/g)	7.64 ± 0.685^c^	22.6 ± 0.631^a^	21.2 ± 0.090^ab^	19.4 ± 0.599^b^	< 0.001
MDA (µg/g)	0.563 ± 0.016	0.527 ± 0.012	0.519 ± 0.0142	0.508 ± 0.147	NS
Blood lipid profile (mmol/L)					
Total cholesterol	2.44 ± 0.147	3.07 ± 0.207	2.97 ± 0.208	2.88 ± 0.154	NS
HDL cholesterol	0.797 ± 0.044	0.644 ± 0.066	0.630 ± 0.052	0.616 ± 0.038	NS
Non-HDL cholesterol	1.64 ± 0.107^b^	2.42 ± 0.175^a^	2.34 ± 0.167^a^	2.27 ± 0.159^a^	< 0.01
Triglycerides	2.84 ± 0.283^a^	2.28 ± 0.179^a^	2.18 ± 0.356^a^	1.60 ± 0.161^b^	< 0.05
Other blood biomarkers					
ALT (U/L)	21.6 ± 3.59^b^	62.2 ± 17.39^a^	40.5 ± 5.82^ab^	72.7 ± 15.1^a^	< 0.05
AST (U/L)	67.3 ± 5.83	99.1 ± 20.21	81.3 ± 7.25	126 ± 20.40	NS
ALP (U/L)	121 ± 5.48^b^	203 ± 17.17^a^	179 ± 10.91^a^	195 ± 7.72^a^	< 0.001
Total bile acids (µmol/L)	0.829 ± 0.105	0.845 ± 0.178	0.803 ± 0.197	0.758 ± 0.248	NS
Glucose (mmol/L)	18.0 ± 1.29	19.1 ± 1.20	20.1 ± 1.31	19.3 ± 1.66	NS

Values are means ± SEMs (*n* = 7). Labelled means in a row without a common letter (a, b, c) differ at *P* ≤ 0.05 (Duncan’s or Dunn’s post hoc test). ALT, alanine transaminase; ALP, alkaline phosphatase; AST, aspartate transaminase. C, control group; HF, high-fat group; HF + PS, high-fat group supplemented with native poppy seeds; HF + DPS, high-fat group supplemented with partially defatted poppy seeds; MDA, malondialdehyde; NS, non-significant. Non-HDL cholesterol was calculated as the difference between the total and HDL cholesterol.

## Discussion

Obesity and obesity-related disorders, such as gut dysbiosis, fatty liver disease or dyslipidemia, are some of the most important health problems for which appropriate dietary changes are needed^20,21^. However, they are often difficult to implement, and additional dietary supplementation may be the way to deal with these problems, provided that an effective preparation is used. This study was intended to answer whether dietary supplementation with poppy seeds may affect the development of obesity-related disorders. For this purpose, we fed rats with a high-fat diet, which was additionally supplemented with 1% native or partially defatted poppy seeds. This level of supplementation translates to a daily consumption of 82 g of poppy seeds (5.5 tablespoons) by an adult human weighing 75 kg, which is the amount that can be consumed during the day. We took this approach to facilitate the translation of the obtained results into further human trials, if needed. The rat model used in this study was characterised by severe metabolic disorders, especially in the liver, which was manifested by overweight, fatty liver and elevated activity of liver enzymes (ALT and ALP).

We used native poppy seeds and their defatted form by cold pressing, which, however, still contained a significant amount of fat (44% vs. 21%) due to the well-known low efficiency of this process^14^. The lack of complete defatting is the limitation of this study, because we were unable to precisely assess the extent to which the lipid fraction of poppy seeds is responsible for their effects. Nevertheless, we believe that partially defatted poppy seeds are such an interesting by-product available on the market that its methodically simplified comparison with native poppy seeds is justified and valuable, especially for practical reasons and the need of their utilization. Moreover, partially defatted poppy seeds had lower percentage of PUFAs in the form of linoleic acid than their native form. Similar relationships were observed by Melo et al.^8^, who compared chemical composition of poppy seeds and their press cake. An explanation for this phenomenon may be that PUFAs can pass from seeds to oil more easily due to their relatively lower melting point and viscosity, which are important factors facilitating cold pressing^14,22^.

In this study, the fibre content was significantly higher in partially defatted poppy seeds than in their native form (33% vs. 20%), and this dietary component is especially important in preventing and managing obesity and obesity-related diseases due to its resistance to digestion, among others^23^. Dietary fibre is fermented by the gut microbiota into SCFAs, including acetate, propionate and butyrate, as the major end products of this fermentation. SCFAs are considered to be indirect nutrients for the body, which, after being absorbed from the distal intestine, participate in the regulation of energy metabolism and fulfil a number of functions. However, not all SCFAs can be beneficial in obesogenic state and, for example, acetate is a substrate for hepatic cholesterol biosynthesis^17^. In the present study, we found the caecal acetate content elevated in rats fed a diet supplemented with native and partially defatted poppy seeds, but it did not significantly correlate with the higher hepatic cholesterol concentration in those animals (*r* = 0.36 and − 0.54, respectively; *P* > 0.05). Moreover, we found a significant reduction of branched SCFAs in the caecum (isobutyrate and isovalerate) by supplementing the diet with partially defatted poppy seeds, but not with their native form. Branched SCFAs are produced in smaller amounts and their effect on the body is insufficiently understood. Nevertheless, both isobutyrate and isovalerate mainly originate from protein fermentation in the distal intestine and thus they are considered as markers of this potentially harmful process for the intestinal epithelium^24^. Interestingly, a negative correlation has been found between the consumption of dietary insoluble fibre and faecal levels of branched SCFAs^24^, which is in accordance with our results, because poppy seeds contain almost exclusively this fibre fraction^8^.

Epididymal fat is an important part of visceral fat in rodents^15^, and in the present study, dietary supplementation with partially defatted poppy seeds prevented its excessive accumulation induced by the diet. In addition, the supplementation with partially defatted poppy seeds lowered triglyceride concentration both in the liver and blood plasma, which did not correlate, however, with the lower *SREBP-1c* mRNA level in the liver (*r* = − 0.25 and –0.61, respectively; *P* > 0.05). SREBP-1c upregulates the expression of genes involved in fatty acid and triglyceride synthesis from non-lipid precursors^18^, so its downregulation in the present study indicates on inhibited hepatic *de novo* lipogenesis by defatted poppy seeds. However, since there were no straightforward correlations, the mechanism behind the aforementioned beneficial effects seems to be more complex. Moreover, these beneficial alterations were not accompanied by a reduction in body weight and body fat percentage nor relative liver mass and liver fat percentage, which was most likely due to similar calorie intake in all HF groups. Unfortunately, the observed decrease in hepatic triglycerides was compensated by higher hepatic cholesterol in both poppy seed-supplemented groups, as mentioned in the previous paragraph in the context of the microbial acetate production. However, the blood cholesterol was not affected by dietary poppy seeds, regardless of their form, whereas the blood triglycerides were lowered by the partially defatted form. Thus, considering the risk for cardiovascular disease, the increased accumulation of cholesterol in the liver found in our study may not be considered as unfavourable for humans. It is hard to speculate which compounds of partially defatted poppy seeds could have been responsible for the observed triglyceride-lowering effects (both in the blood and in the liver); however, these might include phenolic acids, some of which can inhibit hepatic SREBP-1c expression^25,26^. Most likely, PUFAs did not play any role, since native poppy seeds had no significant effects on triglyceride metabolism, and the expression level of *PPAR-α* mRNA did not change either. PUFAs are known PPAR*-α* ligands, thus upregulate the expression of genes involved in the *β*-oxidation of fatty acids^27,28^. We also did not note any significant effects of poppy seeds on the hepatic mRNA level of *PPAR-γ*, which in turn upregulates the expression of genes involved in body’s adipogenesis^28^. Interestingly, in previous studies on rats, whose diet was supplemented with defatted forms of flax and hemp seeds, we also showed some improvements in the lipid metabolism, but those were related to PPAR*-α* and PPAR-*γ*, and not to SREBP-1c^29,30^.

There is a need to recognise alternative, plant-derived sources of components that are usually deficient in the diet. The need is especially important in the context of highly processed food, which contributes to the worldwide pandemic of obesity and diet-related diseases^31^. In the literature, a by-product in the form of defatted poppy seeds is suggested as a valuable source of nutrients, dietary fibre and biologically active compounds^8^. To our knowledge, there have been no nutritional studies available on the health effects of poppy seeds, regardless of their form, despite their interesting chemical composition. Therefore, in the present study, we wanted to fill the gaps aforementioned and verify the potential health consequences of consuming defatted poppy seeds. Our results indicate that a low and regular consumption of partially defatted poppy seeds alters lipid metabolism and may be beneficial in managing obesity-related disorders, such as hyperlipidaemia or triglyceride overaccumulation in the body. As importantly, the study is strictly focused on dietary changes and does not cover all the wide-ranging aspects of obesity development and management, such as social and environmental factors^32^. However, it may indicate a new dietary option for food manufacturers and healthy consumers, and even for people with already diagnosed lipid disorders. The latter should, however, primarily adhere to recognised methods of treatment.

## Conclusions

The rats fed with a high-fat diet were characterised by severe metabolic disorders, especially in the liver, and poppy seeds were unable to prevent them. However, depending on the seed form, the supplementation with the small amount of native or partially defatted poppy seeds differentially affected the microbial and endogenous lipid metabolism in rats fed a high-fat diet. In the distal intestine, both dietary seed forms stimulated acetate production, and the supplementation with partially defatted poppy seeds additionally inhibited the formation of branched SCFAs, which indicates a reduction in putrefaction. Furthermore, both dietary seed forms stimulated cholesterol accumulation in the liver. Only dietary supplementation with partially defatted poppy seeds attenuated visceral fat and hepatic triglyceride accumulations and lowered blood triglyceride levels, and the inhibition of hepatic *de novo* lipogenesis was indirectly involved in this process. These results indicate that a low and regular consumption of partially defatted poppy seeds, a by-product of cold-pressed oil, alters lipid metabolism and may be beneficial in managing obesity-related disorders.

## Methods

### Chemical composition of poppy seeds

Opium poppy (*Papaver somniferum* L.) seeds were purchased in two forms: native and partially defatted by cold pressing. Native poppy seeds were from BioPlanet plc (Leszno, Mazovia Province, Poland), and partially defatted poppy seeds were from Efavit Co. (Poznań, Poland). Both forms were ground for 1 min at a temperature below 37 °C before being used as dietary supplements in the experiment. The chemical composition of native and partially defatted poppy seeds was quantified in triplicate by an accredited testing laboratory (Nuscana, Mrowino, Poland) in accordance with the Polish-European ISO standards, official procedures of AOAC or internal procedures, using commonly known methods. Briefly, the dry matter and ash content were determined by the gravimetric method after drying both forms of poppy seeds at 105 °C and 525 °C (PN-EN 1135:1999), respectively. The total dietary fibre was determined by the enzymatic-gravimetric method (AOAC 991.43:1994). Crude protein was determined by the Kjeldahl method (PN-EN ISO 20483:2014-02), and crude fat was determined by the Soxhlet extraction method. The nitrogen-free extract was then calculated by subtracting water, ash, fibre, crude protein and crude fat from 100. The fatty acid profile of the fat fraction extracted from the native and defatted seeds was determined by gas chromatography with flame-ionization detection after previous conversion of the fatty acids into respective methyl esters (PN-EN ISO 12966-1:2015 and 12966-2: 2011). The chemical composition of native and partially defatted poppy seeds is shown in Table 1.

### Animals, diets and experimental design

The feeding experiment was conducted on 28 male 6-week-old Wistar rats allocated to 4 groups of 7 animals each. Initial acclimatisation of the rats lasted 1 week prior to experimental feeding. The initial body weight of rats was comparable among groups (details in Table 3). For 8 weeks, each group was fed with a modified version of the semipurified diet recommended for rodents by Reeves^33^. The control (C) group was fed a standard diet with regular fat content in the form of rapeseed oil (7% diet), whereas the high-fat group (group HF, positive control) was fed a modification of this diet, in which palm oil and cholesterol were added (18% and 0.5% diet, respectively). In the other two groups, the high-fat diet was supplemented with 1% native or partially defatted poppy seeds (HF + PS or HF + DPS group, respectively). This level of supplementation translates to a daily consumption of 82 g of poppy seeds by an adult human weighing 75 kg, which was calculated on the basis of initial body weight and dietary intake of rats. All dietary modifications were made at the expense of corn starch, and each diet had similar content of nutrients and dietary fibre. The detailed composition of the diets, which were freely available to rats for the entire experimental period, is shown in Table 2. The rats were individually housed in plastic cages and a controlled environment (a 12 h light-dark cycle, a temperature of 21 ± 1 °C, a relative humidity of 50–70% and 20 air changes per hour).

### Body composition analysis

At the beginning and end of experimental feeding, the body lean and fat masses of the rats were determined by time-domain NMR using the Minispec LF 90II analyser (Bruker, Karlsruhe, Germany) according to a previously described method^34^. Based on the body weight and other baseline and endpoint measurements, the body lean and fat percentages and gains were then calculated. The method relies on transmitting various radio frequency pulses into soft body flesh to reorient the nuclear magnetic spins of the hydrogen and then to detect radio frequency signals generated by the hydrogen spins from that flesh. The contrast in relaxation times of the hydrogen spins found among adipose tissue and other soft fleshes is then used to estimate their masses within the body.

### Sampling and analysis of biological material

At the end of experimental feeding, the rats were anaesthetised with a mixture of xylazine and ketamine in physiological salt (10 mg and 100 mg/kg body weight, respectively). Each rat was then weighed, and the abdomen was cut open. Blood was collected from the vena cava into heparinised tubes. The blood was then centrifuged for 10 min at 380×*g* and 4 °C, and the obtained plasma was frozen until analysis. The small intestine, caecum and liver were then removed, weighed and frozen in liquid nitrogen or used for further procedures.

Disaccharidase activities (maltase and sucrase) was measured in jejunal mucosa according to the previously described method of Dahlqvist with modifications^29^. Briefly, an aliquot of mucosal homogenate was incubated at 37 °C with a substrate solution (maltose or sucrose) in a phosphate buffer (pH 7.0). After 15 min of incubation, cold distilled water was added, and the enzymatic reaction was interrupted by immersing the test tube in boiling water for 3 min. A blank with the same composition was simultaneously prepared and immersed in boiling water without prior incubation. Glucose was quantified using a glucose oxidase reagent (Alpha Diagnostic Ltd., Warsaw, Poland), and the disaccharidase activity was expressed as µmol of glucose liberated from the disaccharide per minute per gram of protein. The protein concentration in the mucosa was determined using the Bradford method with bovine serum albumin as the standard.

Samples of fresh caecal digesta were collected, and their pH values were measured using a microelectrode and pH/ION metre (Model 301, Hanna Instruments). The ammonia concentration in the fresh caecal digesta was extracted, trapped in a solution of boric acid and then quantified by direct titration with sulphuric acid in Conway dishes according to the method described by Hofirek and Haas^35^. The short-chain fatty acid (SCFA) concentrations were determined in the caecal digesta after storage at − 20 °C using a gas chromatograph (Shimadzu Co., Japan) and capillary column (SGE BP21, 30 m × 0.53 mm; SGE Europe Ltd.) as previously described^36^.

Liver lipids were extracted according to the method of Folch et al.^37^ with previously described modifications^13^. Briefly, the liver slice was homogenised with a 2:1 mixture of chloroform–methanol using a homogenizer (IKA T25, USA) followed by centrifugation at 15,000×g for 10 min. The supernatant was washed with distilled water, vortexed and centrifuged for 15 min (2500×g). After removing the upper phase, the lower phase containing lipids was evaporated under a nitrogen stream at 37 °C. The lipid fraction obtained in this way was then dissolved with chloroform, and cholesterol and triglyceride concentrations were determined spectrophotometrically in this solution using reagents from Alpha Diagnostics Ltd. (Warsaw, Poland). The hepatic malondialdehyde (MDA) concentration was also determined spectrophotometrically (at 532 nm) using the extraction procedure developed by Botsoglou et al.^19^ and expressed as micrograms of MDA per gram of liver.

The plasma concentration of cholesterol (total and its HDL fraction), triglycerides and glucose, as well as the plasma activity of alanine transaminase (ALT), aspartate transaminase (AST) and alkaline phosphatase (ALP) were determined using a biochemical analyser (Pentra C200, Horiba Ltd., Japan). The plasma total concentration of bile acids was determined using the Cell Biolabs kit (San Diego, CA, USA).

### mRNA quantification

Gene mRNA expression levels in the liver were quantified according to a previously described method^13^ on devices and reagents from Thermo Fisher Scientific (Waltham, MA, USA). Briefly, RNA was extracted from the liver using TRI Reagent solution according to the manufacturer’s instruction. RNA quantity and quality were measured by spectrophotometry using NanoDrop1000 and agarose gel electrophoresis, respectively. cDNA was synthesised from 500 ng of RNA using a high-Capacity cDNA reverse transcription kit with a ribonuclease inhibitor. The beta-actin gene (*ACTB*) was selected as a reference in each particular cases. The mRNA level of each individual gene was determined using the species-specific and recommended TaqMan gene expression assay probe. Amplification was performed using the 7900HT Fast Real-Time PCR System under the following conditions: initial denaturation for 10 min at 95 °C; 40 cycles of 15 s at 95 °C and 1 min at 60 °C. Each run included a standard curve based on aliquots of pooled liver RNA. All samples were analysed in duplicates. The mRNA levels of sterol regulatory element-binding protein 1c gene (*SREBP-1c*) and peroxisome proliferator-activated receptor alpha and gamma genes (*PPAR-α* and *PPAR-γ*, respectively) were normalised to *ACTB* and multiplied by 10.

### Statistical analysis

The results are expressed as the mean ± standard error of the mean (SEM) except for the chemical composition of poppy seeds, whose results were expressed as the mean ± standard deviation (SD). One-factor analysis of variance (ANOVA) and Duncan’s *post hoc* test were used to determine significant differences among groups at *P* ≤ 0.05. If the ANOVA was not homogenous, one-factor Kruskal-Wallis ANOVA by ranks was used followed by Dunn’s Bonferroni-corrected *post hoc* test (*P* ≤ 0.05). All calculations were performed using Statistica version 13.1 (StatSoft Corp., Cracow, Poland). Spearman’s rank correlation was used to measure the relationship between the selected variables with *P* ≤ 0.05 as significant.

### Ethical approval

The experiment protocol was in compliance with European guidelines for the care and use of laboratory animals and it was approved by the Local Institutional Animal Care and Use Committee in Olsztyn, Poland (permission number: 37/2017). This research was also conducted in accordance with the ARRIVE guidelines and regulations. The poppy seeds were purchased as food products and the study complied with institutional, national, and international guidelines and legislation.

## Data Availability

All data generated or analysed during this study are included in this published article.
